# Female Putty-Nosed Monkeys Use Experimentally Altered Contextual Information to Disambiguate the Cause of Male Alarm Calls

**DOI:** 10.1371/journal.pone.0065660

**Published:** 2013-06-05

**Authors:** Kate Arnold, Klaus Zuberbühler

**Affiliations:** 1 School of Psychology & Neuroscience, University of St Andrews, St Mary's Quad, St Andrews, Fife, United Kingdom; 2 Cognitive Science Centre, University of Neuchâtel, Neuchâtel, Switzerland; University of Saint-Etienne, France

## Abstract

Many animal vocal signals are given in a wide range of contexts which can sometimes have little in common. Yet, to respond adaptively, listeners must find ways to identify the cause of a signal, or at least rule out alternatives. Here, we investigate the nature of this process in putty-nosed monkeys, a forest primate. In this species, adult males have a very restricted repertoire of vocalizations which are given in response to a wide variety of events occurring under conditions of limited visibility. We carried out a series of field playback experiments on females (N = 6) in a habituated group in Gashaka Gumti National Park, Nigeria, in which male alarm/loud calls were presented either alone, or following acoustic information that simulated the occurrence of natural disturbances. We demonstrate that listeners appear to integrate contextual information in order to distinguish among possible causes of calls. We conclude that, in many cases, pragmatic aspects of communication play a crucial role in call interpretation and place a premium on listeners' abilities to integrate information from different sources.

## Introduction

Behavioral research on free-ranging primates, while challenging, is the most ecologically valid way to explore the evolutionary origins of human cognitive abilities, including precursors to language. Recently, there has been much theoretical and empirical focus on the extent to which animal vocalizations contain or provide information, and how they might do so. ‘Information’ can be understood as a measure of uncertainty reduction in predicting an outcome of an event [1]. This notion of ‘information’ has been adopted by many researchers in animal communication as a useful concept for understanding the mechanisms by which receivers associate signals with the outcomes of specific events [2], i.e., can make predictions about the future behavior of the signaler or other relevant and imminent events. While animal vocal signals certainly have the potential to provide or transmit information, whether or not they do is still debated [2]–[5]. Calls vary widely in their potential to reliably convey information depending on a number of factors including the degree to which they are acoustically distinct from other call types within any given species repertoire, and also whether they are given in a narrow or wide range of contexts. The alarm calls of some bird and mammal species are regarded as being amongst the most informative because they are considered to be given to a relatively narrow range of external events. This high degree of production specificity has led to the description of such signals as ‘functionally referential’ [6]. For example, the ‘eagle’ alarm calls of vervet and Diana monkeys are reported to be given only when a predatory eagle has been detected. These calls are thought to have high informative value since they are reliable indicators of the presence of predatory eagles (e.g., [7], [8]). In such cases, listeners do not require any additional information in order to select the appropriate anti-predator strategy. These and similar findings are important because they provided evidence that animal calls *might* function as symbols [9], [10], (*c.f.* [11]), the meanings of which are determined solely by associations between the signal and the eliciting event. Such ‘functionally referential’ calls have been found in birds (e.g., [12]), meerkats [13], and primates, including apes (e.g., [14]). However, it is still unclear how common strong signal-event associations are in animal communication. Various studies have shown that predator-specific alarm calls, i.e., acoustically distinct alarm call types that ‘stand for’ different predator classes, are not universally present in primates (e.g., [15], [16]), and some, particularly ground living, species, have graded, i.e., indistinct, alarm calls with low context specificity (e.g., [17], [18]). In these cases, the informative value of calls is low and it is generally accepted that listeners must bear the cognitive burden of extracting information from calls in order to generate meaning [19]. In all cases, a proper assessment of production specificity, which entails systematic recording of all contexts in which the call occurs, is crucial in determining the potential information value of calls, although this has rarely been attempted.

In previous field experiments with arboreal forest primates, male putty-nosed monkeys (*Cercopithecus nictitans martini*) responded to the presence of their two main predators with two loud call types, ‘hacks’ and ‘pyows’. These two call types were most often given as a uniform series of calls (either pure ‘pyow’ or pure ‘hack’ series) or as a ‘transitional’ series (which begin with ‘hacks’, end with ‘pyows’ and appear to be functionally equivalent to pure ‘hack’ series) [20], [21]. However, the two call types can also be organized into a short ‘pyow-hack’ sequence, which instigates group movement in both predatory and non-predatory contexts [22], [23]. In experiments, ‘hack’ and ‘transitional’ series were usually given in response to playbacks of crowned eagle (*Stephanoaetus coronatus*) shrieks and a static crowned eagle model, while ‘pyow’ series were usually given in response to similar leopard-related stimuli [20], [21]. However, the relationship between call series and predator types was probabilistic at best and, more importantly, we also observed that these call series were given in many other contexts that were not related to predators at all. These playback experiments had, therefore, significantly underestimated the full range of contexts in which these calls occurred [24].

Under these circumstances, how do listeners know when calls are given because a dangerous predator has been detected, as opposed to other possible causes? In such cases, focusing on animal pragmatics, i.e., studying the ways in which context contributes to the derivation of meaning, is likely be a more fruitful general approach than looking for symbolic aspects of animal communication [19], [25]. Although this is not a new idea [26], only a small number of studies have looked systematically at the relationship between context and the information content of signals and these have all been carried out on species with graded vocal signals. Baboon responses to playbacks of grunts have been shown to be affected both by the acoustic properties of the calls and the context in which they are presented [27], and prior knowledge about the recent history of social relationships [28]. Baboons also use social knowledge to infer the intentions of callers [29]. More generally, primates appear to be aware of how the vocal behavior of familiar group members relates to their social position. Both chimpanzees and baboons respond more strongly to vocal interactions of group members that are inconsistent with dominance relations than those that are consistent [30], [31].

In this study we have explicitly adopted a pragmatics approach to exploring how primates extract information from what preliminary observations suggested were highly ambiguous, though discrete, signals [20], [21], [24], (see also [32]). We first attempted to record the full range of contexts in which the ‘hack’, ‘transitional’ and ‘pyow’ series of male putty-nosed monkeys were given naturally. While listeners have the opportunity to learn about the range of contexts in which each call series type is produced, it is often difficult to ascertain the specific cause of a calling event, especially in low-visibility tropical rainforests. Previous observations indicated that listeners sometimes attempt to acquire additional information about the behavior of the caller when the cause of calling is not evident [23].

We hypothesized that listeners can disambiguate the cause of the calls by integrating available contextual information. In this study we carried out an experiment on free-ranging putty-nosed monkeys designed to compare the response of listeners to male alarm calls given alone, or paired with additional contextual information. Our playback sequences were designed to mimic natural situations in which the group male either called in response to an audible disturbance, or situations in which listeners had no information about the cause of calls. In a first condition, we presented a series of ‘hacks’. In a second condition, we provided additional contextual information, i.e., the sound of a falling tree just prior to a series of ‘hacks’, so that the cause of calls could be attributed to that event. In a third condition, playbacks of ‘hacks’ were preceded by crowned eagle vocalizations, thereby simulating the presence of a crowned eagle at a particular location. We predicted that monkeys should spend more time looking skywards after hearing hacks alone than in the other conditions in which information about the possible cause of calls was also given, even when this information indicated the presence of a crowned eagle since listeners had been provided with information about the location of the eagle.

‘Pyows’ are associated with a wider range of contexts than ‘hacks’ and are most often given spontaneously [24]. They can also be given to terrestrial predators, including leopards which, though dangerous, are not an immediate threat for arboreal forest monkeys once they have been detected [33]. When hearing ‘pyows’ in the absence of further information, the best strategy for disambiguating the cause of the calls is to monitor the behavior of the caller or of individuals in close proximity to the caller. Therefore, we predicted that if listeners heard only ‘pyows’, monkeys should spend more time looking in the caller's direction than when contextual information was provided in order to gain information about whether he might be calling spontaneously, or in response to a disturbance as indicated by the direction of his gaze and posture, or by the behavior of other individuals that were closer to him.

## Results

### The context of naturally occurring calls

The range of contexts within which different call series types were given and the distribution of call series types across contexts are presented in Table 1. A large number (93%) of natural disturbances which elicited calls included a loud acoustic element, thereby providing listeners with information about the nature of the event. There was a substantial degree of overlap in the contexts in which both call types were given although ‘hacks’ were given in a more restricted range of contexts than ‘pyows’.

**Table 1:** The range of contexts in which males gave ‘hack/transitional’ and ‘pyow’ call series and the frequency with which males were observed to give each call series type within each context.

Context	Call series type			
	‘hack/transitional’ N = 41	%	‘pyow’ N = 127	%
Eagle	7	17.1	*	-
Falling tree/breaking branch^1^	8	19.5	7	5.5
Large terrestrial mammal	-	-	6	4.7
Baboon fight^1^	3	7.3	3	2.4
Flying duck	1	2.4	-	-
Intergroup encounter^1^	-	-	7	5.5
Other male hack/transitional series^1^	22	53.7	21	16.5
Other male pyow series^1^	-	-	22	17.3
Other male P-H sequence^1^	*	-	4	3.1
Spontaneous	-	-	57	44.9

* indicates at least one observation outside the systematic observation period.

1 Note: these events contain a noisy element which informs listeners about the occurrence of the event irrespective of whether it is directly witnessed or not. Observations were carried out over 213 days.

### Responses to male calls and the effect of contextual information

Table 2 shows the time females spent scanning in different directions during the 20 s following presentation of ‘hacks’ alone, and ‘hacks’ preceded by contextual information designed to indicate a likely cause of the male's subsequent calls. In response to stimuli containing ‘hacks’ alone, subjects spent significantly more time looking skywards than when ‘hacks’ were preceded by crowned eagle shrieks or the sound of a falling tree (‘hacks’ alone vs. tree-‘hacks’, *T ^+^* = 21, *N* = 6, *p* = .016; ‘hacks’ alone vs. eagle-‘hacks’, *T ^+^* = 21, *N* = 6, *p* = .016; eagle-‘hacks’ vs. tree-‘hacks’, *T ^+^* = 8, *N* = 6, *p* = .188, post hoc Wilcoxon signed ranks tests). In this series of experiments, time spent scanning in all other directions did not differ significantly according to whether contextual information was presented before hearing ‘hacks’, or not (Table 2).

**Table 2:** Comparison of time spent scanning in different directions following the playbacks of ‘hacks’ presented alone, or preceded by contextual information.

Direction	Stimulus			χ^2^	p
	H	E-H	T-H		
Up	6.17 (4.63)	0.93 (2.42)	0.39 (2.71)	10.18	0.003
Source	5.94 (6.15)	1.97 (3.95)	0.99 (3.45)	4.26	0.136
Down	2.65 (8.99)	0.00 (2.59)	0.08 (2.25)	3.11	0.259
Other	2.09 (3.06)	2.36 (6.51)	7.41 (12.74)	1.83	0.442
Experimenter	2.58 (3.40)	0.00 (1.39)	3.99 (6.78)	3.39	0.201

Medians (and IQ range) for N = 6 subjects and the results of Friedman's analyses of variance. H = ‘hacks’, P = ‘pyows’, T = falling tree, E = eagle shrieks.

In response to stimuli containing ‘pyows’ alone, subjects spent significantly more time looking toward the source of the calls than when ‘pyows’ were preceded by crowned eagle shrieks or the sound of a falling tree (Table 3. ‘pyows’ alone vs. eagle-‘pyows’: *T ^+^* = 21, *N* = 6, *p* = 0.016; ‘pyows’ alone vs. tree-‘pyows’: *T ^+^* = 21, *N* = 6, *p* = 0.016; eagle-‘pyows’ vs. tree-‘pyows’: *T ^+^* = 8, *N* = 6, *p* = 0.188). There was also a tendency for subjects to spend less time looking up after hearing ‘pyows’ alone than if they had first heard eagle shrieks or the sound of a falling tree although this difference did not quite reach significance in pair-wise post hoc tests (‘pyows’ alone vs. eagle-‘pyows’, *T ^+^* = 15, *N* = 6, *p* = 0.031; ‘pyows’ alone vs. tree ‘pyows’, *T ^+^* = 15, *N* = 6, *p* = 0.031; eagle-‘pyows’ vs. tree-‘pyows’, *T ^+^* = 9, *N* = 6, *p* = 0.422, post hoc Wilcoxon signed ranks tests). We found no significant differences in time spent scanning in other directions according to whether or not contextual information was presented before hearing ‘pyows’ (Table 3).

**Table 3:** Comparison of time spent scanning in different directions following the playbacks of ‘pyows’ presented alone, or preceded by contextual information.

Direction	Stimulus			χ^2^	p
	P	E-P	T-P		
Up	0.09 (0.88)	2.58 (4.11)	3.50 (4.51)	6.82	0.034
Source	8.46 (5.76)	0.57 (2.14)	4.37 (9.16)	11.27	0.001
Down	2.50 (7.24)	0.13 (8.21)	0.90 (3.73)	0.40	0.819
Other	3.56 (5.50)	4.43 (7.43)	3.55 (8.55)	1.00	0.740
Experimenter	2.63 (3.05)	2.56 (6.79)	0.49 (1.26)	3.22	0.241

Medians (and IQ range) for N = 6 subjects and the results of Friedman's analyses of variance. H = ‘hacks’, P = ‘pyows’, T = falling tree, E = eagle shrieks.

It could be argued that the reported differences were the result of carry-over effects from responses to the contextual stimuli. We therefore compared the subjects' responses to contextual information alone during the 20 s before the playbacks of the subsequent male calls, and during the 20 s following male calls presented in the absence of preceding contextual, i.e., in response to one stimulus (Table 4). For each stimulus type, we compared the amount of time looking towards the source of the stimulus and found no significant difference. However, subjects tended to look up for longer after hearing ‘hacks’ alone than the other stimuli (‘hacks’ vs. eagle, *T ^+^* = 15, *N* = 6, *p* = 0.031; ‘hacks’ vs. tree, *T ^+^* = 15, *N* = 6, *p* = 0.031, Wilcoxon signed ranks test, two-tailed) and for less time after hearing ‘pyows’ alone than after hearing eagle shrieks or the sound of a falling tree (‘pyows’ vs. eagle, *T ^+^* = 20, *N* = 6, *p* = 0.063; ‘pyows’ vs. tree, *T ^+^* = 15, *N* = 6, *p* = 0.031) though none of these results quite reached significance. There was no difference in the amount of time spent looking up after hearing either of the contextual stimuli alone (eagle vs. tree, *T ^+^* = 7, *N* = 6, *p* = 0.563). No difference was found in time spent looking at the source of the stimulus in any of the conditions.

**Table 4:** Friedman's analysis of variance comparing the median time spent looking upwards, and toward the source of stimuli, following playbacks of male calls alone and contextual stimuli alone.

Direction	Median time spent looking (s)					
	Eagle	Tree	Hacks	Pyows	*χ^2^*	*p*
Up	1.59	1.52	6.17	0.85	15.00	>0.001
Source	4.99	7.17	5.94	8.46	3.00	0.431

We also compared the amount of time spent looking upwards and towards the source of the stimuli within trials in order to investigate whether scanning responses to contextual stimuli affected scanning responses to subsequent male calls, i.e., habituation effects (Table 5). In general, no significant habituation effects were found except in the case of responses to ‘hacks’. Subjects spent less time looking toward the source of ‘hacks’ than the source of preceding contextual stimuli although this result did not quite reach significance for eagle shrieks.

**Table 5:** Comparison of the median time spent looking upwards, and toward the source of stimuli, following playbacks of contextual stimuli and male calls within trials.

Stimuli		Up (s)		*T ^+^*	*p*	Source (s)		*T ^+^*	*p*
Context	Call	Context	Call			Context	Call		
-	Hack	-	6.17			-	5.94		
Eagle	Hack	0.00	0.93	7^a^	0.625	4.48	1.97	1	0.063
Tree	Hack	1.04	0.39	8	0.688	6.13	0.99	0	0.031
									
-	Pyow	-	0.85			-	8.46		
Eagle	Pyow	2.41	2.58	4^b^	0.438	5.58	0.57	2	0.094
Tree	Pyow	1.45	3.50	13^b^	0.188	6.24	4.37	10	1.000

N = 6 except,

a N = 4;

b N = 5 due to ties. α = 0.05.

## Discussion

In previous studies, male putty-nosed monkeys most often responded to the simulated presence of crowned eagles with a series ‘hack’ or ‘transitional’ series, and to the simulated presence of leopards with ‘pyow’ series [18], [19]. However, in the present study, ‘hack/transitional’ series were recorded at least equally often in a variety of other contexts as well, including to non-predatory disturbances and the calls of neighboring males. ‘Pyow’ series were given in an even wider range of contexts, often overlapping with those that elicited ‘hack’ and ‘transitional’ series, and most often without any apparent cause at all. In experiments designed to mimic natural situations in which the group male called in response to an audible disturbance, or situations in which listeners had no information about the cause of calls, we found that listeners spent more time looking upwards in trials that consisted of playbacks of ‘hacks’ alone than those in which ‘hacks’ were preceded by acoustically simulated disturbances indicating a likely cause of the calls. In response to ‘pyows’ alone, listeners spent more time looking toward the presumed location of the caller than when ‘pyows’, similarly, appeared to be given in response to simulated disturbances.

The frequency with which each call series type was observed to be produced in different natural contexts suggests that listeners have sufficient opportunities to form associations between calls and the contexts in which they are given. However, the high degree of overlap in the contexts in which each call series type was heard does not provide the production (context) specificity necessary to form the basis of strong associations between different call series types and any particular class of event, with the exception of eagle detection perhaps. ‘Hacks’ are particularly salient since they function almost exclusively as alarm calls (but see [20], [21]) and are almost always produced in response to crowned eagles at close and mid ranges. However, only seven out of forty-eight recorded ‘hack/transitional’ series were given to eagles. Even if the seven instances in which the context of ‘hack/transitional’ series could not be determined were, in fact, responses to eagles that had not been detected by observers then this would bring the total to fourteen (29%), although confidence that an eagle was not present was high in most cases. This illustrates a further point, that low visibility in a dense canopy can sometimes make it very difficult to see what others can see, and it is likely that not all members of the group will have visual contact with the eagle that the male is responding to in every case, thus reducing opportunities for forming the association even when the cue is present. Given the range of circumstances that elicit ‘hacks’, and the potential danger that only one of them entails, it is crucial that listeners possess some form of mechanism for distinguishing between ‘hacks’ that indicate that an eagle has been detected and those that do not. A falling tree, whilst being noisy and agitating, is not usually threatening and does not require any particular response. However, we recorded eight instances of ‘hack/transitional’ series, and seven of ‘pyow’ series, being given in response to falling trees. Given that both of these call-context parings were experienced as often as ‘hack/transitional series’ were given in response to eagles, the learned associations could potentially be equally strong, thus highlighting the low informative value of the different call series types. Nonetheless, our observations indicated that ‘hacks’ given in series appear to function almost exclusively as alarm calls, while ‘pyows’ do not. In addition, ‘hacks’ and ‘pyows’ are given as part of the short and distinctive ‘pyow-hack sequence’ [20], [21] that is used by males to instigate group movement in both predatory and non-predatory contexts and has no alarm function at all. In this case, the salient context of the calls is the call sequence itself rather than any external stimulus, and is also likely to be a learned association as opposed to requiring semantic/syntactic unpacking of the sequence [34]. In a previous study, analyses of both call types in predatory and non-predatory contexts did not reveal significant structural differences [23].

In our experiments, subjects generally ceased their normal activities after hearing the different stimuli and spent at least 20 s scanning the area. However, the information obtained from each experimental stimulus affected how much time was spent scanning in two possible directions; upwards and toward the source of the stimulus. When listeners heard ‘hacks’ in the absence of any other acoustic contextual information, they spent significantly more time scanning skywards, indicating that they were looking out for something above, than when they were provided with information about the possible causes of calling. It should be noted that when monkeys heard ‘hacks’ under natural conditions they were almost always given in response to something that had an acoustic element, and most often not within visible range, except for the presence of large eagles, which do not make sounds while hunting [35], (S. Shulzt pers. comm., K. Arnold pers. obs.). Our interpretation is that hearing ‘hacks’ in the absence of other forms of acoustic contextual information allows listeners to discount the other possible causes of calls that are associated with this type of call series, infer that the caller may have spotted an eagle, and look upwards in order to attempt to detect it.

In contrast, subjects spent significantly more time looking towards the caller after hearing ‘pyows’ alone than when preceded by contextual information. Our observations of the circumstances surrounding spontaneous ‘pyows’, together with earlier studies indicate that ‘pyows’ function both as alarm calls and also to simply draw attention to the presence and location of the caller [23], [24], [36]. These two functions are consistent with one another given that in a predator context, ‘pyows’ draw listeners toward the location of the caller in order to collectively mob the predator [21], and to make the predator aware that it has been detected [37]. Therefore, when listeners hear ‘pyows’ alone it is not possible to determine whether they are functioning as alarm calls or not. Looking toward the caller is most likely an attempt to gain information about the male’s behavior. Males behave quite differently while producing ‘pyows’ in response to threats as opposed to spontaneously. When calling spontaneously, their attention is not directed to any particular location, nor are they vigilant. This contrasts sharply with situations in which males call in response to a potential threat. In such cases they cease all other activities, become vigilant and, if the object of attention is visible, adopt a distinctive posture, orienting their body toward the disturbance in order to monitor it. If a predator is the cause of the calls, additional contextual information then becomes available even to a relatively distant listener as individuals in close proximity to the caller begin to call themselves as they mob the predator. Listeners looked up for less time after hearing ‘pyows’ given alone than when presented after contextual information, possibly because of the lack of an association between seemingly spontaneous ‘pyows’ and the presence of an eagle close at hand. Listeners also spent a notable amount of time looking toward the caller after hearing ‘hacks’ alone, though less than after hearing ‘pyows’, most probably because hacks are generally given to more serious threats and caller behavior is likely to be a useful source of information in these cases as well.

This study has limitations due to the small sample sizes obtained and so should be interpreted with a degree of caution. Nonetheless, it goes some way to explicate the mechanisms that may be involved in listeners’ ability to disambiguate calls that have multiple or imprecise referents. One previous study has explored a similar theme in relation to grunts given in group movement and infant handling contexts in baboons that have a largely graded vocal system [27]. We hope that this study encourages further work that seeks to replicate our findings in species that have similarly discrete call types. It also highlights the importance of systematically recording natural calling contexts rather than relying solely on experimental techniques that can significantly underestimate the range of contexts in which any particular call occurs.

In conclusion, while much empirical attention has been devoted to highly informative animal vocal signals given to seemingly narrow ranges of events, it is not a ubiquitous feature of non-human primate alarm calling behavior. Graded alarm call systems appear to have low information value [17], [18] but this can also be true of an alarm calling systems that is made up of discrete and easily distinguishable call types [20] as this study has shown. Neither ‘hacks’ nor ‘pyows’ are tightly linked to specific contexts and cannot be said to be particularly informative or meaningful. However, though ‘hack’ series are not context-specific, listeners can infer the presence of an eagle, their most dangerous natural predator, provided that additional contextual information does not indicate an alternative cause of calling. Contextual information is even more important for disambiguating the cause of ‘pyows’ which appear to function primarily as an attention getter but are also given to disturbances [22]–[24]. In such cases, pragmatic aspects of communication play a crucial role and place a premium on listeners’ abilities to integrate information from a number of sources. Calls that lack narrow context-specificity also allow a high degree of flexibility in both call production and comprehension that is absent in context-bound, though potentially more informative, signals.

## Materials and Methods

### Ethics statement

A permit to carry out this research within a protected area was obtained from the Nigerian National Parks Service. Putty-nosed monkeys are not a protected species (rated Least Concern, IUCN)

### Study Site and Subjects

Field experiments were conducted in Gashaka Gumti National Park, Nigeria, between September 2007 and April 2008, by KA together with two field assistants. The study area consisted of primary semi-deciduous lowland rain forest near the village of Gashaka (7°20′N, 11°30′E). Putty-nosed monkeys live in groups of up to 20 individuals comprising one adult male and between 6–9 adult females and their offspring. One group of monkeys, which comprised one adult male, seven females and nine immature individuals during the period of study, had been followed on a daily basis since June 2007 and was habituated to human presence.

### Natural observations of calling contexts

Throughout the study period, we recorded as many natural (i.e., non-experimental) calling bouts given by the male in the habituated group as possible, together with the contexts in which they occurred (N = 240). Calls that appeared to be given spontaneously, i.e., when the male could be observed to be relaxed and the calls were not directed to any particular location, or given in response to an event, were categorized as ‘spontaneous’. However, it was impossible to be absolutely sure that calling was not triggered by an external event that was not detectable by the observers. Calling bouts were excluded from the data set if the context could not be determined with a reasonable degree of certainty, e.g., when more than one contextual factor was available or when the male appeared agitated but the cause could not be determined (N = 72), resulting in a sample of 168 recordings for which the context of calling was known.

### Experimental protocol

From a library of recordings of the calls of the habituated group's resident male, we selected five different examples of pyow series and hack series. We conducted experiments using only recordings of this male's calls as pyows are individually distinctive [38] and the calls of an unknown male at close proximity would have been highly unusual and may have elicited a hostile response. Call stimuli were edited so that each consisted of five calls with a total duration of approximately 10 s.

Contextual information was provided by broadcasting recordings of either crowned eagle shrieks (N = 2; recorded by KZ in the Taï National Park, Ivory Coast) or the sound of a falling tree (N = 2, The Recordist, Creative Sound Design). The experiments were based on a within-subject design. Six playback sequences were played to different subjects no more than once every three days. First subjects were primed with contextual information; either crowned eagle shrieks, the sound of a falling tree, or silence (no discernible context). After 20 s, subjects heard a second stimulus, either a series of five ‘hacks’ or five ‘pyows’. Playback sequences, therefore, mimicked natural situations in which the group male either called in response to disturbances that listeners already had information about, or about which they were ignorant (or possibly spontaneously in the case of pyows).

Subjects were selected by locating a female on the periphery of the group out of visual contact with the male. Each of the six stimulus types were broadcast from approximately 25 m from the known location of the male and at least 50 m from the subject. Playbacks were carried out by a trained field assistant, wearing full camouflage clothing, who continually monitored the male's location while remaining concealed from view. The speaker was positioned 0–2 m from the ground. However, the hilly nature of the terrain in the study group's range allowed broadcast at various altitudes relative to the group which spent much time foraging in river valleys. Subjects' responses were videoed at a distance of between 15–25 m by the experimenter. Subjects had at least partial visibility of >10 m in the direction of both the playback and the experimenter. Trials were never conducted when subjects were in dense foliage. A number of trials were discarded due to subjects moving out of sight before the end of the trial (eagle-hack, N = 7; eagle-pyow, N = 6; tree-pyow, N = 1; hack, N = 1; pyow, N = 4) or if the male vocalized during the video recording period (N = 8). Trials were run until each of the six trial types had been successfully carried out on six females, resulting in a total of 36 trials. Females were individually identified using a combination of phenotypic traits and the presence, or otherwise, of dependent offspring of varying ages. One female was not used as subject because she tended to stay in close proximity to the male and so the >50 m criterion could rarely be satisfied.

### Data analysis

Videos of trials were uploaded onto an Apple iMac for frame-by-frame analysis using iMovie software. Time spent looking in different directions, within the first 20 s of the onset of each stimulus, was recorded for each trial. Looking directions were categorized as: (i) source (in the direction of the speaker), (ii) up (>30°), (iii) down (>30°) relative to the horizontal plane, (iv) at the experimenter, (v) other (in any other direction, i.e., within 30° of the horizontal plane and neither at the source of the stimuli nor the experimenter), (vi) not looking (not visually scanning the area, e.g., manipulating food items, engaged in foraging or social behavior such as grooming). Two randomly selected trials of each type (33% of all trials) were blind coded by a second rater according to written instructions. Cronbach's α test of inter-observer reliability resulted in a score of 0.83 across all trials, indicating reliable coding. We used exact Friedman tests to compare time spent looking in different directions after hearing each male call type broadcast alone or after the presentation of two forms of contextual information. Where significant differences were found, we tested where these differences lay using 1-tailed exact Wilcoxon signed ranks post hoc tests. We consider 1-tailed tests to be appropriate since our specific predictions were informed by previous observations and a pilot study and, in addition, we can think of no plausible mechanisms that would result in the opposite relationships to those predicted. Alpha was set at 0.05 except in post hoc multiple comparisons where a Bonferroni correction was applied resulting in α = 0.0167.
